# Human papillomavirus infection in Shenyang City, People's Republic of China: a population-based study

**DOI:** 10.1038/sj.bjc.6603450

**Published:** 2006-10-31

**Authors:** L K Li, M Dai, G M Clifford, W Q Yao, A Arslan, N Li, J F Shi, P J F Snijders, C J L M Meijer, Y L Qiao, S Franceschi

**Affiliations:** 1 Department of Gynecological Oncology, Liaoning Provincial Tumor Hospital, 44 Xiaoheyan Road, Shenyang 110042, Liaoning Province, China; 2 International Agency for Research on Cancer, 150 cours Albert Thomas, 69372 Lyon cedex 08, France; 3 Department of Cancer Epidemiology, Liaoning Provincial Tumor Hospital, 44 Xiaoheyan Road, Shenyang 110042, Liaoning Province, China; 4 Cancer Institute/Hospital, Chinese Academy of Medical Sciences, 17, South Pan Jia Yuan LN, PO box 2258, Beijing 100021, China; 5 Vrije University Medical Center, Postbus 7057, 1007 MB Amsterdam, The Netherlands

**Keywords:** human papillomavirus, cervical neoplasia, China, epidemiology

## Abstract

To investigate the prevalence of, and risk factors for, cervical infection with human papillomavirus (HPV) in Shenyang City, People's Republic of China, we interviewed and obtained cervical cell samples from 685 women aged 15–59 years enumerated from local population lists. Human papillomavirus DNA was detected in cervical cell samples using a GP5+/6+-based PCR assay for 44 HPV types. Human papillomavirus prevalence was 16.8% overall and 13.6% among women without cervical abnormalities (16.6% and 12.4%, respectively, age-standardised to the world standard population), with no significant trends in HPV prevalence by age group. Of the 32 types identified, high-risk HPV types predominated in all age groups, HPV16 being the most common (3.4% of all women), followed by HPV52 (2.5%) and 58 (1.9%). Multiple-type infections accounted for 31.3% of all infected women. Not being married, reporting multiple sexual partners and husband's extramarital sexual relationships were all significantly associated with being HPV-positive. The disclosure of a relatively high HPV prevalence in Shenyang, in comparison with other worldwide populations, raises important questions concerning the prevention of cervical cancer in China, especially given the promising efficacy of prophylactic HPV vaccines.

Human papillomavirus (HPV) has been established as a necessary cause of cervical cancer, and HPV prevalence in any given population correlates well with cervical cancer risk (Anh *et al*, 2003; Sukvirach *et al*, 2003). The People's Republic of China has historically been considered at relatively low risk for cervical cancer (Parkin *et al*, 2002), but nationwide mortality surveys show a variable pattern of risk across the country (Yang *et al*, 2003a), which is on the increase among younger women, particularly in urban settings (Yang *et al*, 2003b). This phenomenon is probably related to behavioural changes accompanying rapid industrialisation and urbanisation, and concurrent increases of incidence of sexually transmitted diseases (Chen *et al*, 2000). In particular, population mobility and changes in sexual behaviour are expected to have increased the burden of HPV infection. Indeed, a recent study has shown high HPV prevalence in a rural Chinese community known to be at high cervical cancer risk (Dai *et al*, 2006).

In Shenyang City in Liaoning Province, the largest city in north-east China, the population had grown to 7.2 million in 2000, with an annual increase of 7.4% (The fifth national census in 2000, Liaoning Provincial Statistics Bureau Online, http://www.stats.gov.cn). No recent data on cervical cancer exist from the city. The present study aimed to collect data on the age- and type-specific distribution of HPV infection in the urban environment of Shenyang, China, in the framework of a multicentre study coordinated by the International Agency for Research on Cancer (IARC) (Clifford *et al*, 2005).

## MATERIALS AND METHODS

### Study subjects

A list of 1479 women aged 15–59 years was obtained in June and July 2005 from the population list of a residential neighbourhood in Dadong District, Shenyang City. The study purpose was to enrol approximately 100 women in each 5-year age group between 15–19 and 55–59 years. Women were contacted at their home by local administrators and invited for an interview, blood sample collection and gynaecological examination in the study clinic situated in the same district. All mentally and physically competent women aged 15–59 years were eligible for the study, regardless of their marital status. However, gynaecological examination was not carried out on hysterectomised or pregnant women, and was known to be unacceptable to many unmarried women.

Of the 1479 enumerated women, 47 were not found at the address given on the population list, and 41 were not invited as the required sample size for their age group had been reached. Of the 1391 invited women, 391 (28.1%) did not accept the invitation to participate in the study, mainly citing that they did not have enough time or did not think they needed a gynaecological examination. The proportion of nonparticipation was largest (31.2%) among women under 25 years of age. Of an additional 293 interviewed participants who came to the study clinic but refused to undergo gynaecological examination and, hence, a cervical cell specimen collection, 273 (93.2%) were unmarried.

All participants signed an informed consent form according to the recommendations of the IARC and the Cancer Institute of the Chinese Academy of Medical Sciences ethical review committees, which approved the study.

### Study procedures

Study procedures were identical to those reported in a preceding IARC HPV prevalence survey in China (Dai *et al*, 2006). In summary, a standardised risk factor interview was administered to all participants. Then participating women underwent a pelvic examination, and had a sample of exfoliated cervical cells collected for liquid-based cytology and HPV testing. On account of the high proportion of histological confirmation, cervical abnormalities were defined in the present study as presence of histologically confirmed cervical intraepithelial neoplasia (CIN) grade 1 or worse.

Human papillomavirus detection was performed in the Department of Pathology at the Vrije University Medical Center, Amsterdam, the Netherlands, using a GP5+/6+ PCR-based enzyme immunoassay (Jacobs *et al*, 2000). Genotyping of 44 HPV types (HPV 6, 11, 16, 18, 26, 30, 31, 32, 33, 34, 35, 39, 40, 42, 43, 44, 45, 51, 52, 53, 54, 55, 56, 57, 58, 59, 61, 64, 66, 67, 68, 69, 70, 71 (equivalent to CP8061), 72, 73, 81 (equivalent to CP8304), 82 (IS39 and MM4 subtypes), 83 (equivalent to MM7), 84 (equivalent to MM8), cand85, 86, cand89 (equivalent to CP6108) and JC9710) was performed by reverse line blot hybridisation of PCR products (van den Brule *et al*, 2002), and HPV types were classified, for some analyses, into high- and low-risk types (Dai *et al*, 2006).

### Statistical analysis

Human papillomavirus prevalence was standardised by age by applying age-specific prevalence estimates for the age groups 15–24, 25–34, 35–44, 45–54 and 55–59 years to the world standard population reported by Doll *et al* (1966).

Odds ratios (ORs) for HPV positivity and corresponding 95% confidence intervals (CIs) were calculated by means of unconditional logistic regression equations, adjusted for age (15–24, 25–34, 35–44, 45–54, 55–59 years). The statistical significance of trends for ORs was assessed by considering the categorical variables as a continuous variable in the logistic model.

## RESULTS

Of the 707 women who provided cervical cell samples, eight had inadequate cytology results and 14 had *β*-globin-negative samples, leaving 685 women with valid cytology and HPV results, which were included in the analyses that follow. Among them, 29 (4.2%) had histologically confirmed cervical abnormalities, including 19 CIN1, eight CIN2 and two CIN3.

The prevalence of any HPV type was 16.8% (89.7% and 13.6% among women with and without cervical abnormalities, respectively, Table 1). The corresponding prevalences, age-standardised to the world population, were 16.6% overall, and 12.4% among those without cervical abnormalities. In total, 79 women had single-type and 36 had multiple-type infections. In all, 32 individual HPV types were identified.

High-risk HPV types were substantially more frequent (11.7% of all women) than low-risk types (6.4%). The most common types in either single- or multiple-type infections were HPV16 (3.4%), 52 (2.5%) and 58 (1.9%), but HPV type distribution varied by the presence of cervical abnormalities. High-risk HPV types were found in 79.3% of women with cervical abnormalities. The two women identified with CIN3 were HPV16-positive.

**Figure 1:**
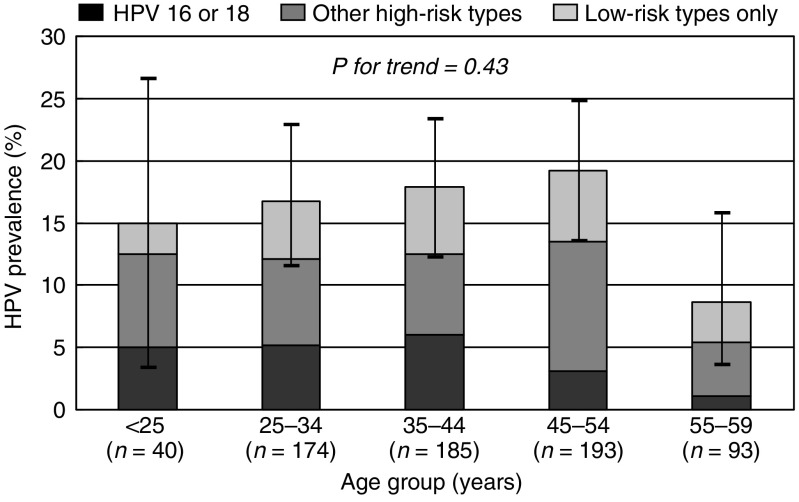
Age-specific prevalence of cervical HPV DNA and corresponding 95% CIs. Shenyang, China, 2005.

Figure 1 shows the age-specific prevalence of HPV, classified hierarchically into (1) HPV 16 or 18, (2) other high-risk types and (3) low-risk types only. Human papillomavirus prevalence was not statistically different across age groups but the lowest prevalence estimate was seen in the 55–59-year age group. High-risk HPV types predominated across all age groups, but HPV 16 and 18 tended to diminish among older women.

Table 2 shows the relationship between HPV positivity and marital status, indicators of sexual behaviour, and use of contraceptive methods, after adjustment for age. Human papillomavirus prevalence was similar among women who were divorced/separated (27.8%) or widowed (25.0%), who showed significantly higher prevalence than married women (OR=2.1, 95% CI: 1.1–3.9). Among only 12 single women who underwent gynaecological examination, three were HPV-positive. There was no significant association between age at first sexual intercourse and HPV positivity. Reporting of multiple lifetime sexual partners (OR_⩾2 *vs* 1 partner_=1.8, 95% CI: 1.1–3.0) and husband's extra-marital sexual relationships (OR=2.5, 95% CI: 1.6–3.7) were significantly associated with HPV positivity. Intrauterine device was the most commonly used contraceptive method (78.4% of study women), followed by condoms (58.1%), whereas only 11.5% of study women reported any oral contraceptive use. There was no significant association between HPV positivity and the use of any type of contraceptive method.

No significant association was found between HPV positivity and education level, occupation, smoking (reported by only 6.4% of women), age at menarche, menopause or first birth, number of births, or history of spontaneous or voluntary abortion (reported by 73.6% of women). Only 44 women reported to have had at least one previous cytological smear (data not shown).

When we evaluated correlates of positivity for high-risk HPV types only, findings were similar to those found for any HPV type (data not shown).

## DISCUSSION

Compared with similar studies in China using comparable methodology, our age-standardised HPV prevalence estimate in Shenyang (17%) is slightly higher than that in the central rural province of Shanxi, known to be at high cervical cancer risk (14%, Dai *et al*, 2006). The HPV prevalence in Shenyang is also similar to that found in high-risk areas for cervical cancer in Latin America (Molano *et al*, 2002; Matos *et al*, 2003; Ferreccio *et al*, 2004) and India (Franceschi *et al*, 2005), although lower than in sub-Saharan Africa (Thomas *et al*, 2004). In the absence of recent data on incidence or mortality of cervical cancer, such high HPV prevalence would suggest a large underlying burden of this disease in Shenyang and similar Chinese populations.

In contrast to previous studies in high-resource countries (Peto *et al*, 2004; Franceschi *et al*, 2006), no decline of HPV prevalence was seen with increasing age, the highest HPV prevalence being among middle-aged women. This age pattern is comparable to that seen in similar survey in rural China (Dai *et al*, 2006), and is also compatible with the flat age-curves found in similar surveys in rural India (Franceschi *et al*, 2005) and sub-Saharan Africa (Thomas *et al*, 2004; Clifford and Franceschi, 2005).

As approximately 80% of HPV infections are estimated to be cleared in 12 months (Molano *et al*, 2003), the majority of the prevalent infections we detected are likely to be recent infections. Therefore, a flat age-curve suggests that in certain populations in developing countries, young women do not acquire new HPV infection more frequently than older women. As a majority of women in our study reported only one lifetime sexual partner, a large proportion of HPV infection at any age is likely to be related to the husband's extramarital sexual relationships.

The reporting of indicators of sexual behaviour, such as number of sexual partners and husband's extramarital sexual relationships were confirmed, as elsewhere (Vaccarella *et al*, 2006), to be the most important determinants of HPV prevalence in Shenyang.

As in most previous population-based surveys (Clifford *et al*, 2005), HPV16 was the most commonly identified type. Confirming findings from the preceding survey in rural China (Dai *et al*, 2006), HPV52 and 58 were more predominant than in non-Asian populations (Clifford *et al*, 2005). These three HPV types were also commonest among women with cervical abnormalities, supplementing the evidence that HPV52 and 58 are over-represented in high-grade squamous intraepithelial lesions and cervical cancer from Eastern Asia compared to other world regions (Clifford *et al*, 2003).

Our present study has strengths and limitations. Among the former, the use of highly sensitive PCR assays and invitation of a representative sample of the general female population. The main limitation of the present study was the lack of complete participation of invited women. However, as HPV infection is asymptomatic, it is unlikely that participation was related to women's HPV infection status. Obtaining cervical cell specimens from unmarried women was confirmed to be difficult (Franceschi *et al*, 2006) and, therefore, the present HPV prevalence should be considered representative of married women only.

In conclusion, in the present absence of data on cervical cancer incidence and/or mortality, the disclosure of a high HPV prevalence would suggest an important underlying cervical cancer burden in Shenyang and similar Chinese populations. The historical picture of China being at relatively low-risk for cervical cancer may not apply to the whole country, and may be changing as a result of the marked behavioural changes accompanying rapid industrialisation and urbanisation. This has implications for the future cervical cancer burden and the priority to be given to preventing cervical cancer in China, especially, given the promising efficacy of prophylactic vaccines against HPV16 and 18.

## Figures and Tables

**Table 1:** Prevalence of HPV types by presence of cervical abnormalities and overall among 685 women. Shenyang, China, 2005

	**Cervical abnormalities**								
	**Absent**			**Present^a^**			**Total**		
**HPV types**	**Single**	**Multiple**	**Total (%)**	**Single**	**Multiple**	**Total(%)**	**Single**	**Multiple**	**Total (%)**
*Negative*			567 (86.4)			3 (10.3)			570 (83.2)
*Positive*									
Any	62	27	89 (13.6)	17	9	26 (89.7)	79	36	115 (16.8)
High–risk	34	23	57 (8.7)	15	8	23 (79.3)	49	31	80 (11.7)
Low–risk	20	19	39 (6.0)	1	4	5 (17.2)	21	23	44 (6.4)
X	6	0	6 (0.9)	1	0	1(3.5)	7	0	7 (1.0)
									
*High–risk*									
16	9	5	14 (2.1)	6	3	9 (31.0)	15	8	23 (3.4)
52	8	6	14 (2.1)	2	1	3 (10.3)	10	7	17 (2.5)
58	1	6	7 (1.1)	3	3	6 (20.7)	4	9	13 (1.9)
18	1	3	4 (0.6)	2	1	3 (10.3)	3	4	7 (1.0)
31	2	4	6 (0.9)	1	0	1 (3.5)	3	4	7 (1.0)
39	2	3	5 (0.8)	0	1	1 (3.5)	2	4	6 (0.9)
56	2	2	4 (0.6)	1	1	2 (6.9)	3	3	6 (0.9)
33	2	3	5 (0.8)	0	1	1 (3.5)	2	4	6 (0.9)
59	3	1	4 (0.9)	0	0	0	3	1	4 (0.6)
45	1	2	3 (0.5)	0	1	1 (3.5)	1	3	4 (0.6)
51	1	1	2 (0.3)	0	2	2 (6.9)	1	3	4 (0.6)
68	1	1	2 (0.3)	0	0	0	1	1	2 (0.3)
35	1	1	2 (0.3)	0	0	0	1	1	2 (0.3)
82	0	1	1 (0.2)	0	0	0	0	1	1 (0.2)
									
*Low–risk*									
42	4	5	9 (1.4)	0	1	1 (3.5)	4	6	10 (1.5)
jc9710	3	4	7 (1.1)	0	1	1 (3.5)	3	5	8 (1.2)
55	2	3	5 (0.8)	0	1	1 (3.5)	2	4	6 (0.9)
66	2	2	4 (0.6)	0	0	0	2	2	4 (0.6)
67	0	3	3 (0.5)	0	1	1 (3.5)	0	4	4 (0.6)
81	2	1	3 (0.5)	0	0	0	2	1	3 (0.4)
11	1	1	2 (0.3)	0	1	1 (3.5)	1	2	3 (0.4)
30	1	2	3 (0.5)	0	0	0	1	2	3 (0.4)
26	1	2	3 (0.5)	0	0	0	1	2	3 (0.4)
40	1	2	3 (0.5)	0	0	0	1	2	3 (0.4)
83	0	2	2 (0.3)	0	0	0	0	2	2 (0.3)
43	0	0	0	1	0	1 (3.5)	1	0	1 (0.2)
53	1	0	1 (0.2)	0	0	0	1	0	1 (0.2)
72	1	0	1 (0.2)	0	0	0	1	0	1 (0.2)
6	1	0	1 (0.2)	0	0	0	1	0	1 (0.2)
54	0	1	1 (0.2)	0	0	0	0	1	1 (0.2)
57	1	0	1 (0.2)	0	0	0	1	0	1 (0.2)
cp6108	1	0	1 (0.2)	0	0	0	1	0	1 (0.2)

a Includes all histologically confirmed CIN1 and worse.

**Table 2:**
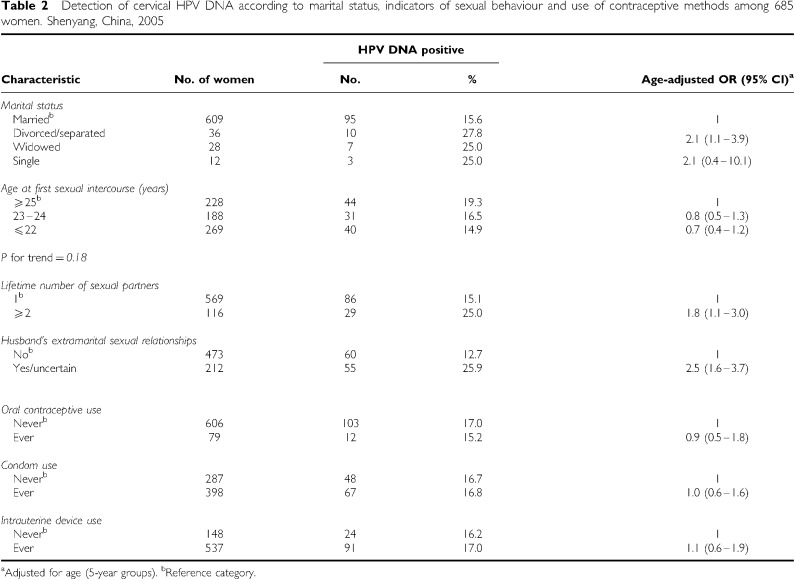
Detection of cervical HPV DNA according to marital status, indicators of sexual behaviour and use of contraceptive methods among 685 women. Shenyang, China, 2005
